# A Perspective on Passive Human Sensing with Bluetooth

**DOI:** 10.3390/s22093523

**Published:** 2022-05-05

**Authors:** Giancarlo Iannizzotto, Miryam Milici, Andrea Nucita, Lucia Lo Bello

**Affiliations:** 1 Department of Cognitive Sciences, Psychology, Education and Cultural Studies (COSPECS), University of Messina, 98122 Messina, Italy; miryam.milici@studenti.unime.it (M.M.); anucita@unime.it (A.N.); 2 Department of Electrical, Electronic and Computer Engineering (DIEEI), University of Catania, 95124 Catania, Italy; lucia.lobello@unict.it

**Keywords:** Bluetooth, wireless passive human sensing, wireless sensor networks

## Abstract

Passive human sensing approaches based on the analysis of the radio signals emitted by the most common wireless communication technologies have been steadily gaining momentum during the last decade. In this context, the Bluetooth technology, despite its widespread adoption in mobile and IoT applications, so far has not received all the attention it deserves. However, the introduction of the Bluetooth direction finding feature and the application of Artificial Intelligence techniques to the processing and analysis of the wireless signal for passive human sensing pave the way for novel Bluetooth-based passive human sensing applications, which will leverage Bluetooth Low Energy features, such as low power consumption, noise resilience, wide diffusion, and relatively low deployment cost. This paper provides a reasoned analysis of the data preprocessing and classification techniques proposed in the literature on Bluetooth-based remote passive human sensing, which is supported by a comparison of the reported accuracy results. Building on such results, the paper also identifies and discusses the multiple factors and operating conditions that explain the different accuracy values achieved by the considered techniques, and it draws the main research directions for the near future.

## 1. Introduction

**Figure 1:**
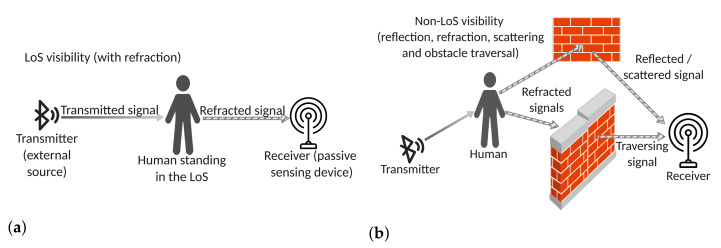
Line of Sight (LoS) visibility (**a**). Non-Line of Sight and through-the-wall (**b**).

In general terms, radio-based passive human sensing refers to the ability to remotely discern the presence and, possibly, the activities, of human beings, without the need for them to bring or wear any device and for the sensing device to emit a probing signal [1]. In fact, the sensing device relies on external sources for the generation of the probing signal by exploiting the radio signal emitted by the most common wireless communication devices (e.g., WiFi and Bluetooth). More specifically, the radio signal is received, processed and the required information is extracted from the deformation and degradation that affect the signal when the presence of a human body obstructs its trajectory (see Figure 1a).

Comparing with conventional, computer-vision based approaches, such as those described in [2,3,4,5], passive remote sensing offers several important advantages. First, there is easier user acceptance, as wireless networks are not able to record images, and therefore, they are not perceived as privacy-invasive by the perspective users. Second, they have wider applicability, as wireless networks do not suffer from Line-of-Sight (LoS) obstruction and bad illumination conditions and may also support through-the-wall sensing. LoS sensing is shown in Figure 1a), whereas non-LoS sensing and through-the-wall visibility are illustrated in Figure 1b).

Finally, deployment efforts and costs may be reduced, as passive sensing may take advantage of the existing wireless communication infrastructures and sensor networks [6], such as the ones used for public utility and safety [7].

In the literature, passive (also called deviceless) remote sensing of human beings was first developed using ad hoc RF transmitters and receivers. Next, WiFi off-the-shelf technology, already successfully applied to a variety of other applications, such as patient monitoring [8,9,10] and factory automation [11], was adopted for this goal. The reason for relying on WiFi mainly resides in the high transmission rate, which allows high-frequency sampling, and in the availability of very detailed Channel State Information (CSI), which allows a stable and reliable extraction of the presence, position and activity information of the human subject. Nevertheless, the Bluetooth technology offers some interesting advantages over WiFi, which suggest the investigation of its application to the passive remote sensing area. In fact, recent studies demonstrated that Bluetooth represents a valid alternative for several reasons:

- Bluetooth is integrated in most portable devices (such as tablet, smartphone, PDA, etc.), and it is often used for personal health monitoring [12,13,14].
- It is energy-efficient (its power consumption is lower than WiFi), in particular after the introduction of the Bluetooth Low Energy specifications in Bluetooth 4.0.
- Its deployment in business, industrial and home environments is simple and flexible, as Bluetooth devices are small, minimally invasive and less expensive than other solutions [15].

Despite the aforementioned advantages, the number of studies that have investigated the application of Bluetooth to passive human sensing so far is significantly lower than the number of works based on WiFi. This consideration motivates this paper, which presents an overview of the most relevant works dealing with Bluetooth-based passive human sensing. The paper provides a thorough analysis of the data preprocessing and classification techniques proposed in the literature, which is followed by a comparison of the reported accuracy results. Building on the presented results, the paper identifies and discusses the multiple factors and operating conditions that explain the different accuracy results achieved by the considered techniques. On the basis of this discussion, the paper draws the main research directions for the near future.

To the best of our knowledge, there are no other reviews, in the literature, dealing with Bluetooth-based wireless passive human sensing at the time of writing this work.

The paper is structured as follows. Section 2 discusses related work on human body passive sensing through wireless communication technologies, with a special focus on Bluetooth.

Section 3 addresses the Bluetooth protocol features that are relevant to the considered application, describing how the human body affects the received Bluetooth signal.

Section 4 discusses data collection, analysis and preprocessing techniques, while Section 5 analyzes the classification methods adopted in the relevant literature.

Section 6 proposes a performance comparison of the results presented in the considered literature, while Section 7 discusses the results obtained by each described technique, deriving insights on the factors that affect their accuracy. Finally, Section 8 provides conclusive remarks and hints for future research.

## 2. Related Work

**Figure 2:**
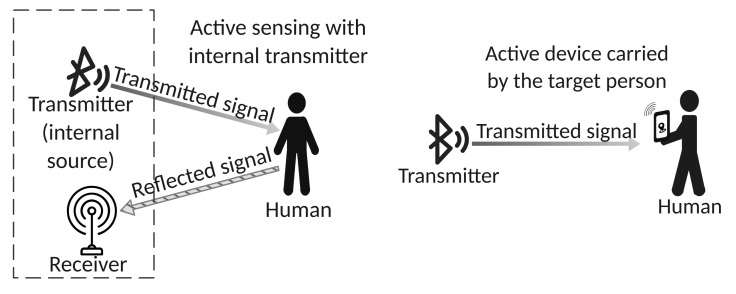
Two classes of active sensing. On the **left**, the sensing device is equipped with both internal probing signal transmitter and receiver. On the **right**, the sensed human brings an active device that receives the probing signal and produces the sensing result.

Most of the literature dealing with Bluetooth indoor signal propagation and the effect of the human body focuses on active (or device-based) indoor localization [16,17,18,19,20,21,22,23,24,25,26,27], where the human to be localized carries, or wears, a Bluetooth device that can track, or can be tracked by, other Bluetooth devices in the environment. This approach belongs to the active sensing class, where either the sensing devices are equipped with both a transmitter and a receiver (see Figure 2 on the left), or the sensed human carries an active device (see Figure 2 on the right).

Device-based sensing approaches can as well be used to detect and track human beings and their activities [28,29]. However, requesting the subjects to carry a traceable device all the time is not always feasible or desirable. Passive, device-free sensing systems, instead, take advantage of the human body’s property of disturbing the radio signals when partially or totally obstructing their trajectory, thus causing signal fluctuations. In principle, by analyzing such fluctuations, it could be possible to separate the influence of the human body from both the original signal and other causes of noise, thus allowing human detection, positioning and activity recognition. However, modeling the “baseline” noise and signal fluctuations that can be expected in the absence of the obstructing human body, thus allowing a reliable detection and tracking, is not a trivial process [30]. The Bluetooth standard stack only allows to measure the Received Signal Strength Information (RSSI), which is a scalar indication of the intensity of the received signal at a given time and is subject to fluctuations and fading during normal operation. The WiFi standard, instead, allows the measurement of the much more informative Channel State Information that, for the most recent devices, is a 3D matrix of complex values representing the amplitude attenuation and phase shift of multi-path WiFi channels [31]. Moreover, the CSI provides more stable information than the RSSI [32,33]. Consequently, the literature regarding the use of WiFi for passive human detection and activity tracking is much wider than the literature on Bluetooth for similar applications.

Human presence can influence a wireless signal in its spatial, temporal and frequency domains [34]. In particular, human motion can affect the frequency and temporal domains, whereas the location of the human body in the environment can affect the signal’s phase. The RSSI alone does not provide phase information, and therefore, some other source of information is needed when the CSI is not directly available. Recently, Bluetooth v5.1 [35] introduced the possibility to measure the signal’s Angle of Arrival and Angle of Departure, which can be used to improve both the positioning and activity tracking accuracy. However, to the best of the authors’ knowledge, such an approach has not been reported in the literature so far.

**Figure 3:**
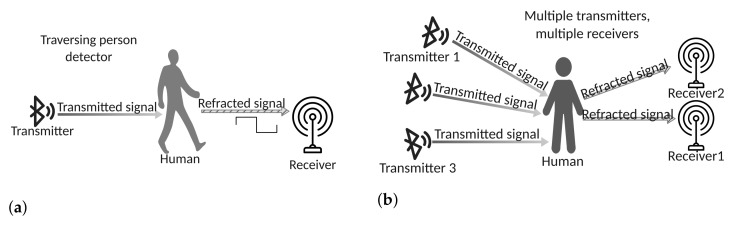
Moving humans counter and multi-transmitter, multi-receiver configurations. (**a**) Moving humans counter. The RSSI undergoes an abrupt change when a human body traverses the signal trajectory. (**b**) Multiple transmitters, multiple receivers.

Multiple RSSI samples can be used to extract more information, either in time or in space. In time, for example, by measuring through a fixed, static receiver a time sequence of RSSI samples emitted by a fixed, static transmitter, it is possible to track human body motion and recognize specific activities (see Figure 3a).

In space, instead, by deploying several transmitters and receivers, a very accurate positioning of a human body can be obtained by exploiting geometrical considerations [36] (see Figure 3b).

**Figure 4:**
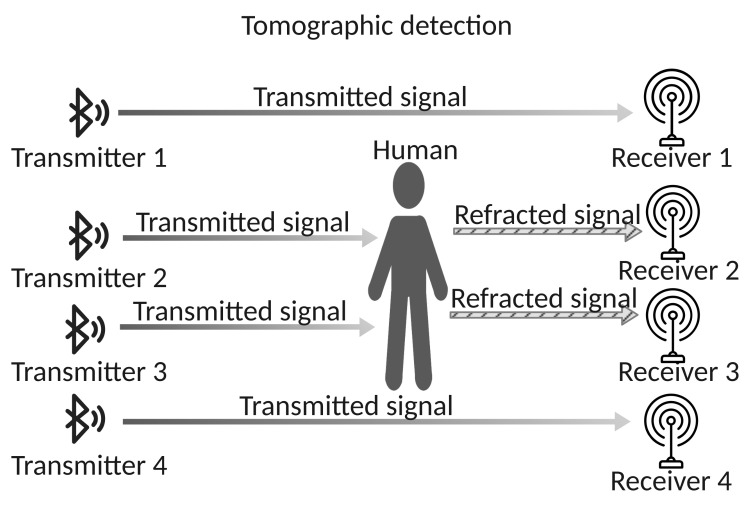
Tomographic sensing.

A special case is based on the radio tomographic approach, where a very high number of transmitter/receiver couples over a spacial grid (see Figure 4) allows to use imaging techniques to obtain an extremely detailed and accurate positioning of the subject [37].

In principle, any wireless transmission technology can support some form of remote human sensing approach. In particular, an interesting comparison is reported in [6], where, curiously, Bluetooth is not mentioned. Table 1 summarizes the main pros and cons of exploiting some of the most popular communication technologies for wireless remote human sensing, such as the ones listed below.

*Radio Frequency Identification* (RFID) is a technology for contactless, short-range, two-way communications that is mainly used for tag detection and identification as well as for low datarate short message exchange. The main advantages of RFID technology are the reasonable resilience to radio frequency noise and the low cost of RFID tags. Conversely, the RFID reader is quite expensive [6]. The main disadvantage of using RFID for passive human sensing is the very short sensing range.

*Frequency Modulated Continuous Wave* (FMCW) radars are instead active sensing devices in which the sensor contains both a transmitter and a receiver [38,39]. The main advantages of applying FMCW to human sensing are the high sensitivity and distance accuracy [6]. However, FMCW-based devices are specifically aimed at sensing, and therefore, a human sensing approach based on this technology cannot leverage existing communication infrastructures, but a separate deployment is needed. Moreover, FMCW-based human sensing is not in the scope of this study, as the focus here is on passive human sensing.

*Bluetooth and its low-energy version* (BLE) are among the most widely adopted wireless communication technologies. Their main advantages are low power consumption, which allows the installation of battery-operated nodes, and the relatively low deployment costs, even in case a specific infrastructure is required. Moreover, such technologies have the ability to support both regular communications and passive human sensing at the same time, as regular advertisement signals are exploited for sensing (see, for example, [40,41]). The main disadvantages of Bluetooth and BLE are that they currently do not provide CSI and an effective way to cope with the abrupt RSSI changes due to Frequency Hopping (see Section 3.2).

*WiFi* is the wireless communication technology that is most widely adopted for passive human sensing. Early works in the literature exploited the WiFi RSSI values, but the most accurate and effective applications are based on the ability of some recent devices to provide Channel State Information (CSI) multidimensional samples, thus supporting a more detailed extraction of human activity information [31]. The main advantages of using WiFi for human sensing are the wide diffusion of the WiFi communication technology and the reliable and fine-grained information provided by the CSI, which allows sensing low-amplitude activities (e.g., hand gestures) and vital signs (e.g., breathing). However, WiFi devices cannot be used for communications and passive sensing at the same time [6], and therefore, a separate infrastructure is needed for sensing or the same infrastructure must be multiplexed in time, thus reducing the time available for both services. Moreover, WiFi power consumption is sensibly higher than Bluetooth power consumption (this is particularly true for Bluetooth Low-Energy), and the deployment cost of an ad hoc infrastructure is also higher. Finally, WiFi is relatively less robust to changes in the environment and noise than other signals [6].

*Visible Light Communication* (VLC) technology is also being exploited for human sensing [42]. VLC technology uses low-cost, high-efficiency photodiodes (LED), is resilient to RF noise, and it can leverage the existing lighting infrastructure. However, such a technology requires a complex light sensing infrastructure that is hardly justifiable by its application to human sensing, as a large number of photodiodes need to be installed on the sensorized environment floor [6].

*LoRa* [43] is a radio frequency transmission technique based on a spread spectrum modulation, which enables long-range transmissions with low power consumption [44]. Both properties are interesting for human sensing. In the work in [45], LoRa is used for active remote human sensing in a radar-like fashion, by equipping the sensing device with both a transmitter and a receiver modified and adapted to the specific application. However, LoRa requires a dedicated infrastructure for sensing.

The *LTE* (long-term evolution) mobile communication system [46], which seamlessly covers almost all areas, both indoor and outdoor, can act as a diffused external illuminator for wireless human sensing [47].

The 6G communication networks also promise localization and human activity detection among their most relevant services [48]. The two main disadvantages of using LTE and 6G for remote human sensing are the long distance between the transmitter, the receiver and the human, which require complex filtering techniques to remove the noise and separate the signals [6], and the significant risk of privacy violation due to both the non-locality of the probing signal and the potential for receiving the sensing signal from a long distance, which would allow for mass monitoring.

One of the earliest and simplest applications of Bluetooth to deviceless human sensing is to support remote elderly care, by allowing the remote caregivers to monitor that their patients regularly take their prescriptions [40,41]. The described system consists of three principal components:

- A Bluetooth beacon positioned under a medicine calendar (i.e., a tool used to norm the assumption of the correct daily dose of drugs).
- A computer tablet positioned at fixed, short distance from the beacon.
- A smartphone for the remote caregiver.

In this application, the Bluetooth beacon periodically transmits a message with a unique ID, while the tablet measures the received signal RSSI. When a human approaches the medicine calendar, thus disturbing the signal sent by the beacon, the RSSI suddenly drops, and the dedicated application running on the tablet records the event and sends a notification to the caregiver’s smartphone. The detection accuracy obtained by the system in a real environment is higher than 90%.

More recently, by measuring and analyzing the fluctuations in the received signal RSSI, a passive device-free system based on a network of Bluetooth Low Energy (BLE) beacons was able to detect the presence [49] and estimate the number [50] of humans in a lecture room. With 24 beacons positioned under the seats in the room and four receivers positioned on the ceiling laterally to the seats, the detection accuracy was higher than 95%, while the accuracy of the number of people estimated was about 82%, with an average room occupancy of about 30 attendees.

In [51], a system to detect the presence of humans in a waiting queue is presented. Usually, waiting queues are controlled by barrier poles with retractable belts, which can be moved if needed, and the system described in [51] is able to detect the transit of people between two barrier poles so as to allow to estimate the queue waiting time. To this aim, the transmitting and receiving devices are positioned on the barrier poles, facing from the two opposite sides of the queue path, and two detection methods are studied:

- Analysis of RSSI variance;
- Analysis of RSSI average.

The results reported in [51] show that the RSSI variance is better suited for detecting the transit of people between the two poles, i.e., the motion of a body traversing the ideal line connecting the two poles (see Figure 3a), while the RSSI average is more suitable for detecting people standing between the two poles. The accuracy of this system in detecting walking people is about 98% using variance and 96% using average. However, the second algorithm is also able to detect motionless people standing in the monitored area, whereas the first algorithm is only suitable for detecting moving people.

## 3. Human Body Influence on the Bluetooth Signal

In order to better understand the advantages and limitations of using the Bluetooth technology for passive human detection and tracking, this section recapitulates some relevant Bluetooth features.

### 3.1. Bluetooth Recapitulation

Bluetooth is a short-range wireless protocol that supports connections within a range from a few meters to a few dozen meters (depending on the antenna gain, the environment and the specific PHY adopted) [52].

The Bluetooth Classic protocol, also referred to as Bluetooth Basic Rate/Enhanced Data Rate (BR/EDR), works in the globally unlicensed Industrial, Scientific and Medical (ISM) 2.4 GHz short-range radio frequency band, which is divided into 79 channels with 1 MHz spacing. The Bluetooth technology is designed to work well even in very noisy environments, copying with fading and interference. To this end, Bluetooth uses the Frequency Hopping Spread Spectrum (FHSS) [53] technique, that forces two connected devices (master and slave) to frequently change the dedicated communication channel, so the devices hop from one channel to another according to a pseudo-random sequence. The channel sequence is maintained by the master device through a map in which the channels are marked as ‘in use’ if they work properly and ‘unused’ otherwise. This map is updated after a channel is found working well for a given time interval and is shared with the secondary devices so as to have the same information at both ends of the communication.

Two types of Bluetooth connections are available:

1. Asynchronous Connection-Less: a uni-directional communication, in which a slave device (also called advertiser or broadcaster) periodically sends packets, while a master device (also called hub or scanner) continuously scans the channels while waiting for packets.
2. Synchronous Connection-Oriented: a bi-directional communication, in which a connection between a master and a slave is established over a dedicated channel.

The most relevant advantages of the Bluetooth technology are the resilience to noise and interference, thanks to the FHSS technology, the low cost of the devices, and the low power consumption compared to other technologies. In particular, the introduction of Bluetooth Low Energy (BLE) has further reduced the power consumption, thus improving the lifetime of battery-powered devices. For this reason, BLE is also widespread in Internet of Things (IoT) applications.

#### 3.1.1. Bluetooth Low Energy

Bluetooth Low Energy divides the ISM band into 40 channels with 2 MHz spacing. Three channels are used as advertising channels (or primary channels) for broadcasting, while the remaining 37 data channels (or secondary channels) are used for data exchange after a connection event. Mobile devices can be designed to support both Bluetooth Classic and BLE (dual-mode device) or to only support BLE (single-mode device). Single-mode devices are generally used in applications that require low power consumption as a major constraint. Moreover, the BLE stack is thinner compared to the Bluetooth Classic one, to reduce the firmware footprint and protocol management complexity for the applications that run directly on sensors [54].

The first version of BLE protocol was introduced with Bluetooth v4.0, and then, it was updated up to Bluetooth v5.3.

Comparing with the previous versions, in Bluetooth v5.0, major improvements were introduced. First, the coverage range was extended from about 50 m to more than 200 m for outdoor environments, whereas in indoor environments, the range changed from about 10 m to about 40 m. Furthermore, the Bluetooth Core Specification v5.0 [55] introduced a new way to perform advertising, called Extended Advertising, which allows the 37 channels previously reserved for data communication to be also used as secondary advertising channels. Traditional advertising transmits the same payload on the three primary channels, whereas the Extended Advertising transmits payload data only once on a secondary channel. This way, the total amount of transmitted data is lower, and therefore, the duty cycle is reduced. Another benefit of the Extended Advertising is that using a secondary channel to transmit the payload, 255-byte-long packets can be broadcast. In the previous versions of the protocol, instead, only 31-byte-long packets could be broadcast. With this version of BLE, it is also possible to chain packets together and transmit each chained packet on a different channel.

Bluetooth v5.0 also introduced the Periodic Advertising, which allows the receivers to synchronize their scanning for packets with the schedule of the transmitter device. In the previous versions, the advertising process included a degree of randomness in the timing of the advertising packet transmission to avoid repeated packet collisions. However, this implies that scanners could lose some packets, i.e., those transmitted out of their round of scan. In the Periodic Advertising, scanning is performed within the transmission window of the transmitter, thus avoiding such packet losses, and therefore in a more power-efficient way.

In addition, Bluetooth v5.0 reduced the minimum allowed Advertising Interval from 100 ms to 20 ms, thus allowing a rapid recognition of the advertising beacons.

Bluetooth v5.1 [35] introduced a new feature that allows Bluetooth devices to determine the direction of a Bluetooth incoming transmission.By equipping either the receiver or the transmitter with an array of antennas, the receiver can determine either the Angle of Arrival (AoA) or the Angle of Departure (AoD), respectively [56].

In both methods, a special signal, called a direction-finding signal, is transmitted by the transmitting device and used by the receiving device to calculate the direction of the received signal, which in ideal conditions corresponds to the direction along which the transmitting device lays. In the AoA method, the receiving device (that is connected to an array of antennas) receives different copies of the same signal from different consecutive antennas in the array. The received signals are phase-shifted due to the different distances of the receiving antennas to the single transmitting antenna. The Angle of Arrival θ is computed from the phase difference according to Equation (1), where λ is the wavelength, ψ is the phase difference and *d* is the distance between two consecutive antennas in the array [56].

(1) $$\theta =arccos(\frac{\psi \lambda }{2\pi d})$$

In the AoD method, instead, the transmitting device is equipped with an antenna array which emits a signal from each of the antennas. The receiving device, which is equipped with a single antenna, given that λ is the wavelength and *d* is the distance between two consecutive antennas in the transmitting array, determines the phase difference ψ from two received signals and computes the direction θ according to Equation (2).

(2) $$\theta =arcsin(\frac{\psi \lambda }{2\pi d})$$

In Bluetooth 5.1, the direction-finding signals are generated by both defining a new Link Layer Protocol Data Unit (PDU) for direction finding between two connected devices, and a way to use the existing advertising PDUs for connectionless direction finding. In both cases, a special field, known as the Constant Tone Extension (CTE), is added to the end of the PDUs.

Bluetooth v5.1 also introduced the Randomized Advertising Channel Indexing, which allows the devices in advertising state to randomize the selection of advertising channels so that they are not selected in strict order (37, 38, 39) as in the previous Bluetooth versions but in a random order. This feature improves packet collision avoidance.

In Bluetooth v5.2 [57], the LE Power Control feature was introduced. This new feature allows the transmitting devices to dynamically change their transmission power level and inform the receiving device that this has happened. This process is useful to keep high the signal quality and low the error rates, respectively. Moreover, the process also improves coexistence in general, thus benefiting all the protocols working in the 2.4 GHz frequency band.

Bluetooth v5.3 [58] introduced some improvements in the Periodic Advertising, i.e., the host of a receiving device can inform the controller that a packet has been found to contain data that has already been received in an earlier packet. The controller can, therefore, disregard the current periodic advertising packet and immediately switch to another channel.

### 3.2. Influence on Bluetooth Signal

Numerous studies proved that radio signals in the 2.4 GHz frequency band are easily influenced by the human body. An experiment explicitly aimed at demonstrating the effects of a human body occluding the Line-of-Sight between a transmitter and a receiver (see Figure 1a) was reported in 2014 [59], where the received signal power was measured with and without obstruction, and the measured attenuation was approximately 10 dB. However, signal fluctuations were exploited to detect the presence of people in an environment already in 2006 [60].

Received signal fluctuations can be caused by changes in the environment, in the state of the transmitter or the receiver, or by design, e.g., because the protocol requires actions that produce such fluctuations. In order to exploit the signal fluctuations for passive human sensing, it is mandatory to exclude the unwanted causes or, at least, to find a way to separate their effects from the effects of the targeted human body. Compared to WiFi, Bluetooth appears to be better suited for this purpose, because it is less subject to electromagnetic noise thanks to the FHSS technique. However, the FHSS itself by design generates fluctuations in the received signal due to the communication channel switch. In fact, measurements at the receiver side of the signal emitted by a Bluetooth beacon in advertising mode showed that different channels have different noise and attenuation characteristics [20]. To reduce the influence of the channel hopping on the signal RSSI, each Bluetooth channel should be modeled and processed separately [16]. In the reported experiments, four BLE beacons were used. In particular, three beacons were modified to allow a separate RSSI measurement for each advertising channel, while the other one was not modified. After measuring the RSSI values of all beacons, the RSSI variance was calculated for each beacon. The modified beacons allowed the calculation of separate variances for each channel, while the non-modified beacon only allowed the calculation of the variance of the hybrid signal resulting from the channel hopping. As the Bluetooth protocol does not allow the determination of the time instant at which the hopping takes place, and it does not report the current advertising channel, it was not possible to filter out the RSSI fluctuations produced by the hopping. Consequently, the hopping contributed to the overall RSSI variance. As it was expected, the RSSI variance calculated over the signal transmitted by the unmodified beacon was significantly larger than the per-channel RSSI variances obtained by the modified beacons.

The described study demonstrated that the FHSS causes a significant increase of the RSSI fluctuations, which would heavily impact the reliability of a passive human detection approach based on a set of unmodified Bluetooth devices. For this reason, most of the research works in this area rely on some way to manage each advertising channel separately.

## 4. Data Preprocessing

The demonstration that the wireless signal generally suffers from the noise due to the surrounding environment is introduced in [61]. Moreover, endogenous fluctuations, i.e., the ones coming from inside the device, always affect the received signal. The aim of the preprocessing stage is ideally the elimination, and more in general the attenuation, of noise and endogenous fluctuations, so that the only fluctuations present in the signal after this step are those generated by the human presence between the connected devices.

In [62], a Kalman filter was used for signal noise reduction. The Kalman filter uses linear models, i.e., linear transformations both in the transitions from the current state to the next state and in the transformation from state to measurement, and it also assumes that the noise associated to both the measurements and state of the system is Gaussian. Unfortunately, the hypotheses of linearity and Gaussian noise are not always satisfied in wireless passive human sensing, as shown in [61], where the Kalman filter and the Moving Average filter were compared.

In [62], the Kalman filter performed well with the available data, whereas in [61], such a filter performed poorly, flattening the signal to the point where relevant peaks corresponding to the presence of a human body were deleted. The Moving Average filter, instead, is simpler and less accurate than the Kalman filter under linearity and Gaussian noise hypotheses, but it performed better on RSSI data [61].

An advanced version of the Moving Average filter, i.e., the Exponential Weighted Moving Average filter, was adopted in [22]. Unlike the Moving Average filter, this filter assigns different weights to the values of the considered time series according to an approximated exponential law. The filtered RSSI value is computed according to Equation (3), where α∈[0,1] is the smoothing factor. In particular, the value of α in [22] is 0.05.

(3) $$f_{i,j}\left[n\right]=\alpha RSSI_{i,j}\left[n\right]+\left(1-\alpha \right)f_{i,j}\left[n-1\right]$$

A similar principle is used in [51], where data preprocessing consists of computing the weighted average according to Equation (4), where α=0.9 produces a larger weight for the previous values than for the current value, thus reducing the effect of noisy outliers.

(4) $$Mean_{current}=\alpha Mean_{old}+\left(1-\alpha \right)RSSI_{current}$$

In [63], a passive detection system is described, in which the signal is preprocessed using an α-trimmed mean filter. The α-trimmed filter is generally used in very high noise conditions. In this case, calculating the mean of the signal as the average of the samples is not recommended, because an outlier might significantly alter it. Instead, given a sliding window with *q* RSSI values, this filter sorts the RSSI values and then removes α extremes. After this process, the average of the remaining values is computed according to Equation (5), where 0≤α≤0.5.

(5) $$f\left(q;a\right)=\frac{1}{q-2\lceil \alpha q\rceil }\sum_{i=\lceil \alpha q\rceil +1}^{q-\lceil \alpha q\rceil }RSS_{i}$$

The described process has an offline phase, which produces an estimate of the initial parameters of the system, and an online phase, which produces an estimate of the detected human bodies location, according to the received RSSI samples. Since the environment may significantly change between the offline estimation phase and the online phase, the Analysis of Variance (ANOVA) [64] is adopted to check if the estimate of the initial parameters is still valid.

In [16], a localization system is discussed that uses BLE beacons with a modified firmware able to select a specific channel (either 37, 38, or 39) to send the advertising packets through. This way, one of the causes of increased RSSI variance is removed, but a new problem is introduced. In fact, some packets may get lost in some channels during a scan round due to the fact that the transmitter might send its packet out of the receiver scan interval. At each scan round, the receiver stores the RSSI values from each channel in a vector of RSSI values that represents the RSSI sample for that round. If a packet is lost, the vector has one component missing, and this leads to errors in the analysis steps. As a consequence, some method to fill the missing component of the vector is needed. First, the missing sample component must be detected, and then, an adequate value must be selected to replace the missing value. The chosen solution is that the missing sample component is replaced by the median value of the last $WL_{1}$ samples from the same channel.

Other preprocessing methods based on statistic techniques are available in the literature. The works in [49,50] describe a passive system that is able to detect the presence, and count the number, of human beings in a lecture room. To train and test this system, three datasets were used:

- DS1, containing raw RSSI values.
- DS2 and DS3, containing preprocessed data.

The first dataset was produced by collecting five measurement sessions for each lecture, thus obtaining a total of 80×5=400 sessions. The installation was composed of 24 transmitting beacons and four receivers. Provided that each beacon sent one frame per second, and that each session lasted 300 s, for each session, a total of 24×4×300 = 28,800 samples were collected. To reduce the dataset size, a summarized dataset DS2 was built by calculating, for each measurement session, four features, i.e., mean, variance, trimmed-mean, and trimmed-variance, which were calculated according to Equation (5). Next, the position of the beacons was taken into account by introducing a weighting factor depending on the receiver–transmitter distance. The weighting factor was a normalization matrix *W*, which is used to apply a Min/Max normalization to the data. The set of normalized data was the third dataset DS3. Experimental results show that with the described preprocessing approach, the performance improved from 70% to 98%.

Recently, Deep Neural Networks are being applied also to passive human sensing. The main advantage of deep learning techniques is that they do not need, in general, to manually build specific features for the classification process. Instead, such features are calculated by a neural network that is adequately trained over large amounts of data [65], thus consistently reducing the amount of preprocessing. In [66], for example, raw RSSI values are simply averaged over one-minute intervals and then fed to a deep neural network that is trained to output an estimate of the initial and final position of a human moving in the monitored area.

## 5. Classification Methods

The lack of protocol support to gather the Channel State Information (CSI) sensibly reduces the amount of information that can be obtained by analyzing the received signal. Most likely for this reason, to the best of the authors’ knowledge, as reported in Section 6, the current literature regarding human body sensing through Bluetooth mainly focuses on indoor passive people detection, counting, and approximate motion tracking, whereas complex activities recognition and vital signs sensing have not yet been investigated.

For the considered applications, statistic techniques, Machine Learning techniques, and Artificial Neural Networks are adopted in the literature, as described in the following subsections.

### 5.1. Statistical Methods

The approaches defined as “statistic” use methods based on the analysis of either the mean or the variance of the RSSI values. In [51], two algorithms based on these techniques are presented. The first algorithm, based on the analysis of the variance of a stream (sequence) of RSSI values, is more suitable to detect the motion of a human body, for example, when entering an area of interest. In this case, the RSSI values are detected in subsequent time instants, and therefore, their sequence represents the evolution of the Bluetooth signal perturbation when a human body moves in the area between the receiver and the transmitter devices.

In general, the RSSI variance is calculated over a sliding window always containing the last *n* samples according to Equation (6), where $x_{i}$ represents the current RSSI value and μ is the average of all the RSSI values within the sliding window.

(6) $$Var\left(x\right)=\frac{1}{n}\sum_{i=1}^{n}{\left(x_{i}-\mu \right)}^{2}$$

The size *n* of the sliding window must be chosen according to the specific application. If *n* is too large, too old RSSI values are considered in the calculation, and this could lead to delayed detection. If *n* is too small, the smoothing effect of previous samples on the calculation is not sufficient to remove the measurement noise, thus leading to false positives or false negatives in the classification. In [51], the optimal value of *n* is 10. It was determined considering the time it takes a human to cross the monitored area and the number of messages received for second. Moreover, the mean μ in Equation (6) is replaced with a weighted average computed according to Equation (4).

In the second algorithm reported in [51], the weighted average calculated according to Equation (4) is subtracted from each new RSSI sample and, if the resulting value exceeds a given threshold $T_{m}$, then a detection event is triggered. The weighted average is not updated when a detection event is triggered, because the average is intended to represent the “background” condition, where no human body is present in the monitored area.

The threshold $T_{m}$ is critical and requires a complex calculation involving a preliminary calibration, which is repeated every time something changes in the system deployment, and an initial stabilization time interval that is needed every time the system is turned on or reset. Moreover, the formulation of $T_{m}$ includes some constants that were experimentally determined on the basis of the available data and setup and that might potentially undermine the generality of the proposed approach.

### 5.2. Machine Learning Methods

Machine Learning techniques include both classification and regression algorithms, so they can be used both for presence detection and counting people. For example, K-Means and Gradient Boosting were adopted in [67], whereas Artificial Neural Networks, regression models and Decision Trees (Random Forest) were adopted in [68], and Random Forest were applied in [69]. Passive, BLE-based presence detection systems in the literature exploit Logistic Regression, k-Nearest Neighbor (KNN), and Support Vector Machine (SVM) with linear, polynomial and Radial Basis Function kernel. In [49], a presence detection system was developed, and the algorithms mentioned above were compared on three different datasets. Such datasets, here called DS1, DS2, and DS3, were built according to the process formerly described in Section 4, i.e.,

- DS1, containing the raw RSSI data.
- DS2, containing data resulting from the first preprocessing step.
- DS3, containing data resulting from the second preprocessing step.

The best accuracy was obtained with the (SVM) algorithm [70] using a Radial Basis Function (RBF) kernel applied to the third dataset. An RBF kernel [71] is a function used when the boundaries of the classes are hypothesized to be curve-shaped and nonlinear. An RBF kernel involves the choice of two hyperparameters, i.e., the penalty parameter and the kernel width. In [49], such parameters are auto-optimized in the SVM implementation. In 98.97% of cases, this algorithm recognized the presence of a subject between two nodes. The KNN algorithm, instead, was more efficient with the first and second datasets and obtained higher accuracy than the other algorithms.

In general, all the analyzed algorithms performed similarly when applied to the same dataset, apart from a few exceptions. In particular, for the dataset DS1, the highest accuracy obtained was 70.15% with the KNN algorithm, while the Logistic Regression algorithm obtained 69.81%. If we look only at the accuracy that the two algorithms achieve, the KNN performed better than the Logistic Regression, but the KNN is a parametric algorithm in which the execution time increases with an increasing amount of data. The Logistic Regression algorithm, instead, is a linear algorithm and is faster than the KNN. The SVM algorithm performed slightly worse than the other algorithms on DS1, especially with the linear kernel (62.67%). The reason for this could lay in the distribution of the data. The linear SVM algorithm assumes that a linear boundary can be found between two classes, whereas the kernel trick supports different, nonlinear models of the data. However, in some cases, even the nonlinear kernels struggle in approximating the strongly nonlinear distribution of real data.

For the dataset DS2, containing four features for every transmitter–receiver couple, the KNN resulted as the best performing algorithm with 96.90% accuracy, whereas the SVM with an RBF kernel produced 96.46% accuracy.

The situation changed for dataset DS3, which was normalized to keep in account the distance between the transmitter and the receiver. The SVM with the RBF kernel performed better than the other algorithms (98.97%).

**Figure 5:**
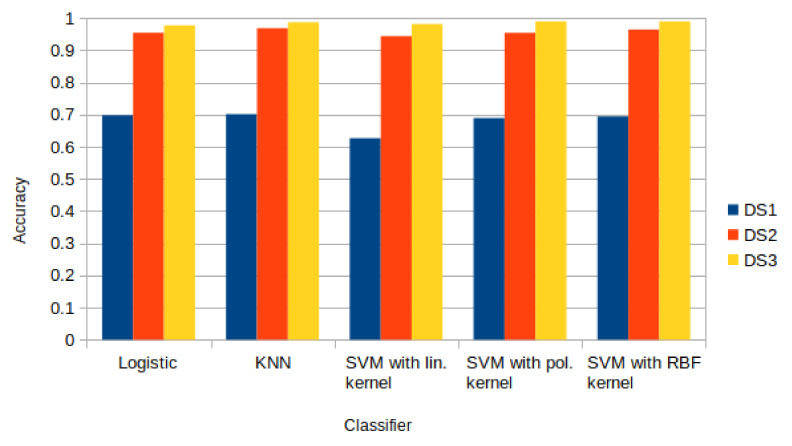
Comparison of the classification models used in [49] with the datasets DS1 (without any processing), DS2 (after aggregation and preprocessing), and DS3 (after normalization with weight factors).

An analysis of the accuracy obtained by applying the described algorithms to all the datasets evidences that the preprocessing stage is always useful to increase the classification accuracy. In fact, as it is shown in Figure 5, the classification accuracy obtained with DS1 is 70%, with DS2 approaches 97%, and with DS3 approaches 99%.

**Figure 6:**
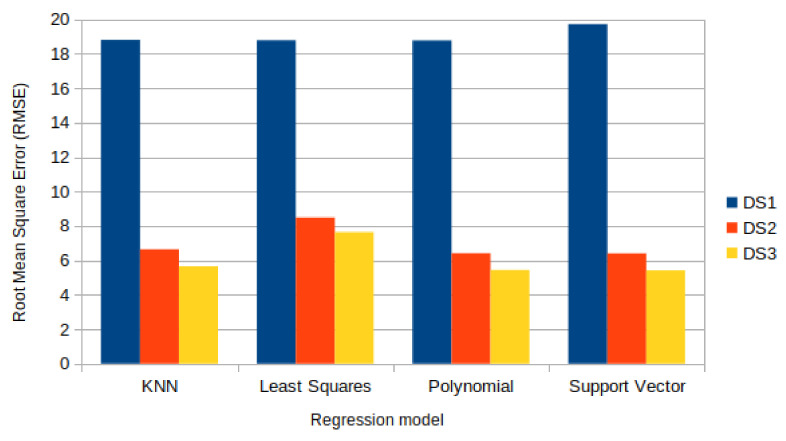
Comparison of the regression models used in [50] with datasets DS1 (without any processing), DS2 (after aggregation and preprocessing), and DS3 (after normalization with weight factors). The error measure is the Root Mean Square Error (RMSE).

The regression algorithms used to develop the counting system, instead, are the KNN Regression, the Least Squares Regression (LSR), the Polynomial Regression, and the Support Vector Regression. These algorithms were compared in [50], in which a counting system was developed and the same preprocessing approach of the previous paper was adopted, obtaining similar performance improvements. In fact, for the dataset DS1, the lowest Root Mean Square Error (RMSE) among all the considered approaches was 18.78, for the second dataset DS2, it was 6.40, and for DS3, it was 5.42. Notably, for DS2 and DS3, the LSR algorithm performed considerably worse than the others, as shown in Figure 6.

As a consequence, the selection of the best algorithm requires further considerations regarding the resource requirements or other specific characteristics of the intended application. In particular, the computational complexity of the considered approach, and therefore its execution time and memory requirements, can sensibly influence the choice of the approach, depending on the hardware that will be used for implementing the system.

### 5.3. Artificial Neural Networks

Artificial Neural Networks are still little used in Bluetooth-based passive counting and detection, whereas the development of a Bluetooth-based localization system exploiting Artificial Neural Networks is described in [66].

In [18], a system for obstruction detection between two nodes, aimed at RSSI signal correction, was developed. Two Artificial Neural Networks were compared to detect obstructions, i.e., a Multi-Layer Perceptron (MLP) network, with two hidden layers of 20 neurons, and a Radial Basis Function (RBF) network, with only one layer of 20 neurons. The comparison demonstrated that both the neural networks detected the obstructions between nodes with high accuracy, with the MLP performing better (91%) than the RBF (89%) according to an estimate based on the data reported in the paper. The detection rate of the proposed approaches in discriminating clear Line-of-Sight, i.e., when no human body was present in the LoS between transmitter and receiver, was also measured. The MLP obtained a 94% detection rate, while the RBF obtained a 92% detection rate.

## 6. Overall Performance Comparison

Table 2 provides a comparison of the different approaches addressed in this paper. Presence detection refers to methods able to reveal the presence of a human body either still or in motion, whereas motion detection refers to methods that only reveal moving human bodies. People counting, instead, refers to methods able not only to reveal the presence of a human body but also to count the number of human bodies present in the monitored area. Finally, clean Line-of-Sight (LoS) detection refers to approaches able to detect the presence of a human body obstructing the line of sight between the transmitter and the receiver (see Figure 1a).

The approaches discussed in this section were trained and tested on different datasets and with different sensors set up. As the codebase and the datasets of the cited studies are not publicly available, the comparison is based on the data reported in the cited works.

The comparison shows that the accuracy of all the techniques is above 87%, and in some cases, it approaches 100%. Nevertheless, some significant differences need to be taken into account. In [40], a simple statistical approach was used for human detection, i.e., when the value of the average RSSI was above a threshold, a detection event was triggered. With this approach, the detection rate was slightly above 90%. Conversely, in [51], a more complex statistical approach was used, and two algorithms were implemented based on the RSSI-variance and RSSI-mean. The first algorithm is more suitable to detect a human crossing the LOS between two nodes, and it obtains an accuracy of 98%. The second algorithm, instead, is best suited for detecting a human that stands between two devices, and it obtains an accuracy of 96%.

In [49], different Machine Learning algorithms for human detection were compared. The best result was obtained with the SVM algorithm using an RBF kernel (98.97%) on a set of preprocessed data. However, the other algorithms perform similarly on the same type of preprocessed data (≈98%). On raw RSSI data, all the tested algorithms performed worse, with accuracy values between ≈70% and 62%. As stated in Section 5, this result supports the observation that appropriate preprocessing is generally needed to improve the accuracy of the classification and regression algorithms and reduce the size of the final dataset, thus shortening the execution time of more complex algorithms, such as the k-Nearest Neighbor one. In [50], a very similar approach was applied to human counting, obtaining an RMSE of 5.42.

In [18], Artificial Neural Networks were used to detect a human body obstructing the space between a transmitter and a receiver, although the system was indeed intended for the correction of the RSSI signal in case of obstructions. The proposed networks, an MLP and an RBF, reached 91% and 89% accuracy, respectively, thus underperforming compared to the statistical approaches. However, the proposed Artificial Neural Networks were only tested on raw data. In principle, they could benefit from data preprocessing, thus further improving their performance. Consequently, more experiments are needed to better understand which approach has the higher potential over the others.

## 7. Discussion

In the previous sections, some applications of the Bluetooth technology (in particular, BLE) to passive human detection, counting and tracking in indoor environment were described and analyzed. Moreover, a comparative overview of their performance was provided.

As the signal RSSI depends on the position and distance between two Bluetooth devices and on the geometric and physical characteristics of the environment, the development of a “generic” passive human sensing system able to cope with all the possible variations in the environment and spatial deployment of the transmitting and receiving devices would require a generalization ability that is hardly obtainable.

Current approaches, instead, rely on some training or initialization process that builds a model of the received signal RSSI that is able to explain all the possible fluctuations of the RSSI values, thus allowing their classification. Such a model can be either explicit, e.g., defined in terms of statistical parameters, or implicit, e.g., obtained by training an ANN.

Building a data-driven model, however, requires that a suitable dataset is gathered from the specific environment where the system will be deployed; i.e., a long data collection process is needed to initialize the system. Furthermore, if Neural Networks are exploited, the data collection process becomes longer and longer, because large datasets are needed to train Neural Networks. The lack of universal datasets to train and test the developed systems makes the comparison of existing techniques difficult.

Another issue often highlighted in the literature is the impossibility of independently extracting the RSSI signal values from each advertising channel of the BLE beacons. The BLE beacons need to be modified at the hardware or firmware level in order to transmit on a certain preset channel and to allow the researcher to discriminate the variation in the signal due to the presence of a human body from other fading effects.

According to Table 2, the RSSI-variance and RSSI-mean approaches used in [51] might be considered as the best performers for presence detection. The reported accuracy results are even better than those reached by more recent Artificial Neural Networks approaches. However, in this study, the BLE transmitters were modified to transmit only on one advertising channel, so that the channel information can be transmitted to the receiver into the payload of the advertising message. Such a modification allows to separately analyze each advertising channel, thus substantially reducing the variance in the RSSI signal and dramatically improving the detection stability and reliability. However, the proposed modification, although being frequently adopted in the relevant literature, is not allowed by the Bluetooth standard, and therefore, it represents a substantial violation of the protocol. In other words, the proposed approach would not work with off-the-shelf beacons.

The Machine Learning techniques used in [49] obtained about 98% accuracy. In particular, as already stated in Section 5, 98% accuracy was reached by using the SVM algorithm with an RBF kernel on a reduced and normalized dataset. In this case, unlike the previous approach, only standard devices were used, and only four receivers were used with 24 transmitters, instead of requiring the same number of receivers and transmitters. As a consequence, the approach proposed in [49] can be applied to a much wider range of cases and environments while performing close to best in class.

## 8. Conclusions

This paper provided a reasoned overview of the Bluetooth-based approaches for passive remote sensing of the human body reported in the literature. The work illustrated and commented on the pros and cons of each approach. Moreover, the experimental results and performance of the considered approaches were described, compared and discussed.

Although the inherent limitations imposed by the Bluetooth protocol may affect its applicability to passive human sensing, a number of recent research works proposed specific preprocessing and non-parametric classification and regression techniques, and they demonstrated the effectiveness and accuracy of the proposed approaches. In general, the adoption of BLE-based remote passive wireless human sensing approaches should be preferred whenever the power consumption of the sensing devices, the deployment cost, and the simultaneous communication and sensing capability are very relevant. However, some interesting aspects still need to be investigated. In particular, the FHSS mechanism significantly affects the reliability of RSSI-based human sensing, and therefore, several works in the literature address modifications of the transmitting device original firmware in order to either bypass the FHSS mechanism or include the channel information in the transmitted frame. Such modifications, however, violate the Bluetooth protocol and should be avoided, also because bypassing FHSS reduces the robustness to noise of the proposed approach. A possible alternative approach can be to estimate, at the receiver side, the transmitter channel mapping and, therefore, to foresee the next hop. Effective techniques are available to obtain such an estimate, such as the ones in [72,73,74].

Furthermore, the recent introduction in the Bluetooth standard of the direction-finding technology has a potential for supporting the development of novel approaches able to extract more information from the BLE signal, thus allowing for more accurate and detailed human sensing applications, similarly to what happened with WiFi. In particular, when determining the Direction of Arrival (DoA) of the received signal (see Section 3.1.1), the receiver can compute a suitable estimate of the phase shift of the reflected, scattered and refracted signal components, and leverage such information to produce a more accurate sensing, thus allowing for complex activity recognition, vital signs detection, and multi-person tracking.

## Figures and Tables

**Table 1:** Pros and cons of the most relevant applications of wireless communication technologies to wireless remote human sensing, as reported in the literature.

Wireless Technology	Advantages	Disadvantages
RFID	Resilience to RF noise	Very short range
FMCW	High sensitivity; High distance resolution	Active approach; Ad hoc infrastructure
Bluetooth, BLE	Widespread use for communications; Low power consumption; Low deployment cost; Simultaneous communication and sensing	No support for CSI; Abrupt changes in RSSI due to FHSS
WiFi	Widely adopted for communications; High spatial resolution; Reliability	Not all devices provide CSI; Non-simultaneous communications and sensing; Relatively high deployment cost; Relatively high power consumption
VLC	Resilience to RF noise; Relatively cheap sensing devices	Complex ad hoc sensing infrastructure
LoRa	Long sensing range; Low power consumption	Active approach
LTE, 6G	Wide availability of the illuminating signal; Stability and reliability	Complex noise filtering and signal separation techniques; Severe privacy concerns

**Table 2:** Performance comparison of the considered approaches.

Application	Preprocessing	Classification/Regression Techniques	Accuracy
Presence detection [40]	-	Statistical techniques	90%
Presence detection [51]	Weighted Moving Avg.	RSSI-mean	96%
Motion detection [51]	Weighted Moving Avg.	RSSI-variance	98%
Presence detection [49]	α-trimmed filter and normalization	Logistic Regression, KNN, SVN (linear, polynomial and RBF kernel)	≈98%
People counting [50]	α-trimmed filter and normalization	Least Squares Regression, NN Regression, Support Vector Regression, Polynomial Regression	±5.42 miscalculation ^1^
Presence detection [18]	-	Neural Networks: MLP and RBF	(MLP) 91%, (RBF) 89%
Clean LoS detection [18]	-	Neural Networks: MLP and RBF	(MLP) 94%, (RBF) 92% clean LoS detection rate ^2^

^1^ This value is not an accuracy value, but the Root Mean Square Error. In [50], such an error metrics was chosen because it represents the misestimation in counting the number of people in the room. ^2^ These percentages are detection rates and not accuracy values. Accuracy is the sum of the true positives and true negatives over the whole set of samples, whereas here, the true positives over the whole set are considered.

## Data Availability

Not applicable.
